# On the Operando Structure of Ruthenium Oxides during the Oxygen Evolution Reaction in Acidic Media

**DOI:** 10.1021/acscatal.3c01607

**Published:** 2023-05-19

**Authors:** Nipon Deka, Travis E. Jones, Lorenz J. Falling, Luis-Ernesto Sandoval-Diaz, Thomas Lunkenbein, Juan-Jesus Velasco-Velez, Ting-Shan Chan, Cheng-Hao Chuang, Axel Knop-Gericke, Rik V. Mom

**Affiliations:** † Leiden Institute of Chemistry, Leiden University, 2300 RA Leiden, The Netherlands; ‡ Theoretical Division, Los Alamos National Laboratory, Los Alamos, New Mexico 87545, United States; § Lawrence Berkeley National Laboratory, 1 Cyclotron Rd, Berkeley, California 94720, United States; ∥ Fritz Haber Institute of the Max Planck Society, Faradayweg 4-6, 14195 Berlin, Germany; ⊥ National Synchrotron Radiation Research Center (NSRRC), Hsinchu 30076, Taiwan; # Department of Physics, Tamkang University, No. 151, Yingzhuan Rd, New Taipei City 25137, Taiwan

**Keywords:** operando XAS, ruthenium oxide, oxygen evolution reaction, electrocatalysis, O K-edge XAS, Ru M-edge XAS, Ru L-edge XAS

## Abstract

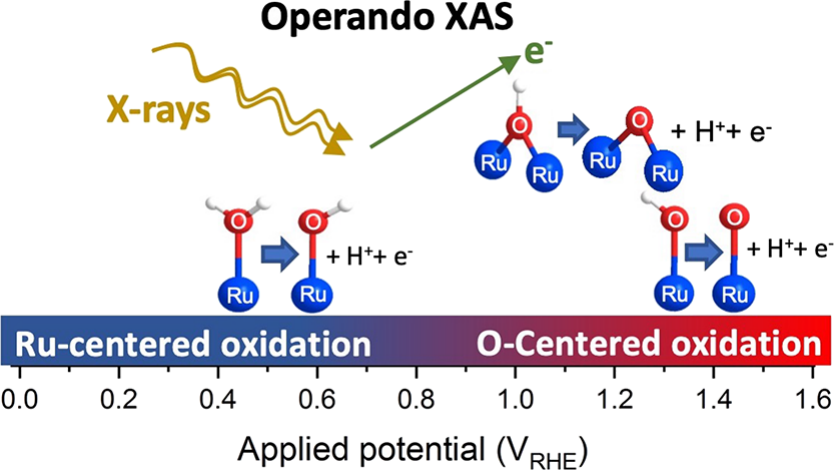

In the search for rational design strategies for oxygen evolution reaction (OER) catalysts, linking the catalyst structure to activity and stability is key. However, highly active catalysts such as IrO_*x*_ and RuO_*x*_ undergo structural changes under OER conditions, and hence, structure–activity–stability relationships need to take into account the operando structure of the catalyst. Under the highly anodic conditions of the oxygen evolution reaction (OER), electrocatalysts are often converted into an active form. Here, we studied this activation for amorphous and crystalline ruthenium oxide using X-ray absorption spectroscopy (XAS) and electrochemical scanning electron microscopy (EC-SEM). We tracked the evolution of surface oxygen species in ruthenium oxides while in parallel mapping the oxidation state of the Ru atoms to draw a complete picture of the oxidation events that lead to the OER active structure. Our data show that a large fraction of the OH groups in the oxide are deprotonated under OER conditions, leading to a highly oxidized active material. The oxidation is centered not only on the Ru atoms but also on the oxygen lattice. This oxygen lattice activation is particularly strong for amorphous RuO_*x*_. We propose that this property is key for the high activity and low stability observed for amorphous ruthenium oxide.

## Introduction

The oxygen evolution reaction (OER) serves as the anodic half reaction in key technologies for sustainable chemistry, such as electrocatalytic water splitting,^1,2^ electrochemical ammonia synthesis,^3^ the conversion of CO_2_ to hydrocarbons,^4,5^ rechargeable metal-air batteries,^6^ and regenerative fuel cells.^7^ In many of these technologies, the OER is a major source of catalyst degradation and energy loss.^8,9^ As a result, the search for active and stable OER catalysts is one of the biggest topics in the field of electrochemistry. Although this has led to a variety of catalysts suitable for alkaline electrolytes,^10−13^ the oxides of iridium and ruthenium have proven hard to beat for the industrially more desirable acidic electrolytes.^14^ For example, in the field of green hydrogen production, alkaline^15−17^ and near-neutral^18^ electrolyzers use nonprecious transition metal compounds based on Co, Mn, Ni, and Fe, whereas the current state-of-the-art PEM (proton exchange membrane) electrolyzers employ Ir- and Ru-based catalysts. However, the scarce availability of iridium and ruthenium necessitates extremely efficient use of these materials. Hence, (rational) optimization of iridium- and ruthenium-based catalysts is of critical importance to enable their widespread use in industrial applications.

To design efficient water splitting catalysts, an important step is to identify which aspects of the catalyst structure dictate their activity and stability. Several observations provide a “smoking gun” in this direction. For example, ruthenium oxides are found to be more active but less stable as compared to iridium oxides.^1,19,20^ Similarly, amorphous oxides are more active yet less stable during the OER than their crystalline counterparts.^21−23^ These observations point at a link between the activity and stability of these materials. This could occur, for example, when the OER and catalyst dissolution reaction share one or more intermediates.^24−26^ For the development of ruthenium and iridium oxide-based catalysts, this leads to the question of whether activity and stability can be optimized independently or whether one can at best achieve a compromise between activity and stability.

Understanding the operando structure of ruthenium and iridium oxides, including catalytic intermediates, may shed further light on this question and improve our understanding of the reaction mechanism. Catalysis on IrO_*x*_ and RuO_*x*_ is generally considered to occur at coordinatively unsaturated sites (CUS sites), where the lattice Ru and O coordination lies below the optimal six- and three-fold coordination, respectively.^27^ Such sites appear to be activated for the OER through oxidation. For example, in situ Ru L_3,2_-edge X-ray absorption spectroscopy (XAS)^28^ on amorphous RuO_*x*_ indicates that the average oxidation state of the ruthenium sites changes from Ru^3+^ to Ru^4+^ when the applied potential is increased from 0.25 to 1.05 V_RHE_ (potential with respect to reversible hydrogen electrode), in agreement with the oxidation peaks observed in the cyclic voltammogram of RuO_*x*_. Similar studies^29−33^ on IrO_*x*_ electrodes also indicate oxidation of the metal sites. However, the oxidation that activates the IrO_*x*_ for the OER is not only centered on the undercoordinated Ir atoms. Operando O K-edge XAS showed that the OH groups on the coordinatively unsaturated oxygen sites are oxidatively deprotonated to form electrophilic O^((1+δ)−)^ species.^33−36^ Molecular dynamics simulations indicated that this electrophilic character activates the oxygen atoms for O–O coupling,^37^ which is an essential step toward the formation of O_2_. Interestingly, the density of O^((1+δ)−)^ species in the oxygen lattice has an important impact: when O^((1+δ)−)^ species are in close proximity, this lowers the energy barrier for the O–O coupling step.^37^ As a result, amorphous IrO_*x*_ with many unsaturated oxygen sites is significantly more active than crystalline IrO_2_ with few defects.^36^

On the basis of the results from studies on iridium oxide, it seems plausible that the high activity and low stability of ruthenium oxide during the OER may be linked to the redox behavior of its oxygen lattice. However, little is known about the electronic structure of oxygen atoms in ruthenium oxides at OER relevant potentials. Here, we fill this gap by directly probing the reactive oxygen species in ruthenium oxides during the OER. We study the behavior of the surface and subsurface oxygen atoms in ruthenium oxides at potentials below and at the onset of the OER. Using operando O K-edge XAS, we have probed the redox reactions of the oxygen atoms at applied potentials between the open circuit potential and the OER regime. The corresponding changes in the oxidation states of surface and subsurface ruthenium atoms are mapped out using Ru M_3_- and L_3,2_-edge XAS, whereas the morphological evolution is followed using electrochemical scanning electron microscopy (EC-SEM). When coupled with density functional theory (DFT) calculations, the experimental spectra capture how the oxide is activated for both OER and catalyst dissolution.

## Experimental Methods

### Sample Preparation for XAS

**Figure 1:**
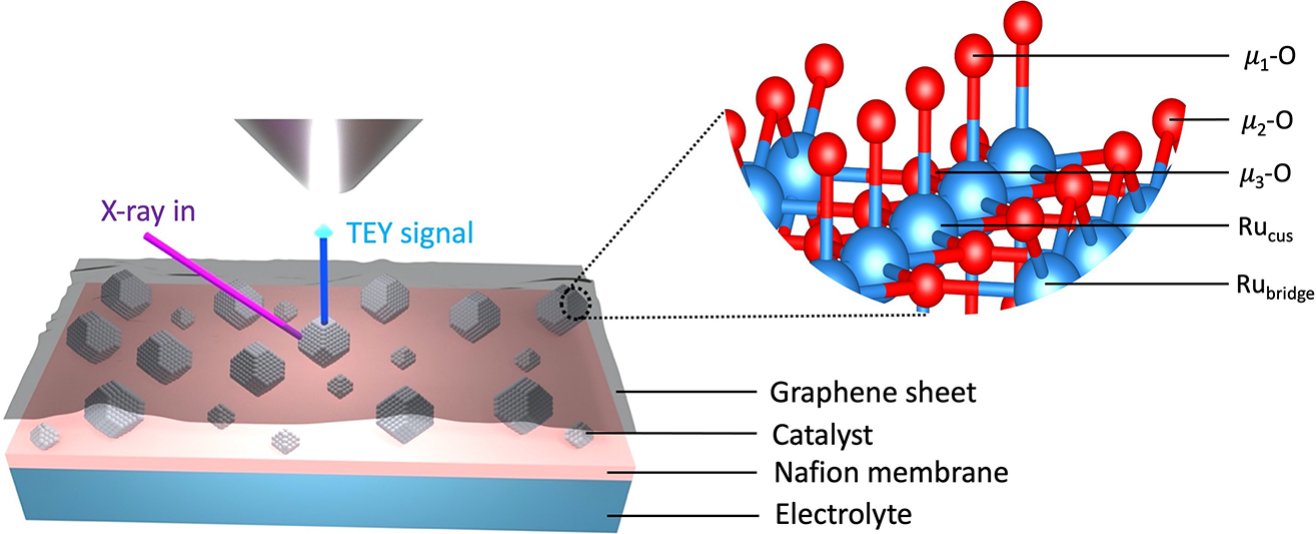
IEM spectroscopy cell assembly. Schematic representation of the confined electrolyte approach. The ball and stick model shows the three types of oxygen coordination and two types of ruthenium coordination that can be found in ruthenium oxides, exemplified here for the case of the rutile RuO_2_(110) surface.

For this study, we used the ion exchange membrane (IEM) cell and SiN_*x*_ cell developed at the Fritz Haber Institute.^38^ Complete details of the cell assembly and sample preparation can be found in Sections S1 and S2 of the Supporting Information (SI). Briefly, to prepare amorphous RuO_*x*_H_*y*_ films for the IEM cell, metallic ruthenium was sputter deposited on a commercially available Nafion 117 membrane using a Cressington sputter coater (0.1 mbar Ar, 40 mA plasma current), resulting in a film thickness of 10–20 nm. Bilayer CVD graphene purchased from Graphenea was transferred on top of the sputtered metallic film, which resulted in a sandwiched assembly as schematically represented in Figure 1. Metallic ruthenium was converted to amorphous RuO_*x*_H_*y*_ films in the spectroscopy cell via electrochemical oxidation. From the existing literature,^27,39^ it is well known that repeated anodic cycling of metallic ruthenium leads to the formation of an amorphous oxide layer. Fifteen potential cycles were applied in the range 0.0–1.35 V_RHE_ at 50 mV/s in 0.1 M H_2_SO_4_ followed by chronoamperometry at 1.25 V_RHE_ for 45 min and 20 more potential cycles. Note that in this potential range, dissolution of RuO_*x*_ is not a factor.^14^ The resulting amorphous ruthenium (hydr-)oxide films were used to record O K-edge and Ru M_3_-edge XAS and Ru 3d XPS spectra.

The sandwiched assembly for polycrystalline RuO_2_ films used in the IEM cell was prepared by a Na_2_SO_4_ transfer method. In this method, metallic ruthenium is sputter deposited on a Na_2_SO_4_ crystal that is converted to polycrystalline RuO_2_ by calcination at 400 °C followed by transferring the RuO_2_ layer onto a Nafion substrate and finally covering the assembly with bilayer graphene. Previous studies^40−43^ have shown that the variation in calcination temperature of a ruthenium precursor leads to oxides of different crystallinity. A calcination temperature of above 350 °C forms the rutile structure of polycrystalline ruthenium oxide. The detailed synthesis procedure is documented in SI Section S2.

### Catalytic Testing with RDE

The OER activity of the amorphous and polycrystalline ruthenium oxide was evaluated using a three-electrode rotating disc electrode (RDE) setup, which employs a reversible hydrogen electrode (hydroflex from Gaskatel) as the reference electrode and a platinum wire as the counter electrode. The RDE electrodes were prepared in a similar manner as the working electrodes for the operando XAS cells. For the synthesis of RuO_*x*_, 20 nm metallic ruthenium was sputter deposited on a polished titanium substrate using a Cressington 208HR sputter coater (0.1 mbar Ar, 40 mA plasma current). Fifteen potential cycles were applied in the range 0.0–1.35 V_RHE_ at 50 mV/s in 0.1 M H_2_SO_4_ followed by chronoamperometry at 1.25 V_RHE_ for 45 min and 20 more potential cycles. To synthesize polycrystalline ruthenium oxide, the 20 nm sputter deposited metallic ruthenium films (on titanium substrates) were calcined at 400 °C for 2 h. The OER activity was measured by linear sweep voltammetry (LSV) from 1 to 1.5 V_RHE_ at 5 mV/s while rotating the electrodes at 1600 rpm.

### Operando X-ray Spectroscopy

The spectra were obtained at two different synchrotron facilities. The operando O K-edge and Ru M_3_-edge XAS and Ru 3d XPS spectra were recorded at the ISISS beamline of the BESSY II synchrotron facility in Berlin, Germany. The Ru L_3,2_-edge spectroscopy was performed at beamline 16A1 of the National Synchrotron Radiation Research Center (NSRRC) in Hsinchu, Taiwan.

The O K-edge and Ru M_3_-edge spectra were recorded in total electron yield (TEY) mode using the IEM cell.^35^ As displayed in Figure 1, the ruthenium oxide electrocatalysts are sandwiched between a proton exchange membrane (PEM) and a bilayer graphene sheet with electrolyte flowing underneath the PEM. The catalyst is wetted by the electrolyte that diffuses through the PEM. The graphene cover confines the electrolyte vapors, thereby acting as a barrier between the catalytic section and the vacuum while remaining transparent to photoelectrons. Ru 3d XPS spectra were collected using the same confined electrolyte approach, employing the ambient pressure X-ray photoelectron spectrometer (APXPS) available at the ISISS beamline. Each spectrum was recorded on a fresh spot to avoid beam damage effects.

The Ru L_3,2_-edge spectra were recorded in total fluorescence yield (TFY) mode in the SiN_*x*_ cell. In this type of cell, the catalyst layer is deposited on a 100 nm thick SiN_*x*_ X-ray window, which separates the wet electrochemical environment from the vacuum in the spectroscopy chamber. Detailed descriptions of the methodologies have been published elsewhere.^38,44,45^ The spectra in both the IEM and SiN_*x*_ cell were obtained while holding the potential constant at the indicated value.

### Operando Electron Microscopy

Electrochemical scanning electron microscopy was performed on a modified scanning electron microscope (FEI Quanta 200 FEG) according to the methodology described by Falling and co-workers.^45^ The sample was prepared by sputter deposition of metallic ruthenium on a commercially available Fumatech FAD55 membrane for 80 s at 0.1 mbar Ar, 40 mA plasma current using a Cressington sputter coater. A sandwiched assembly was created by transferring a bilayer CVD graphene obtained from Graphenea on top of the sputtered catalyst, as shown schematically in Figure 1. The operando flow cell consisted of an Ag/AgCl reference electrode and a platinum wire as counter electrode. The images were recorded at an acceleration voltage of 5 kV. To demonstrate the oxidation of ruthenium oxides by electrochemical cycling, the first EC-SEM image was recorded at OCP before exposing the catalyst to any electrochemical treatment. The next images were recorded at OCP after the catalyst was oxidized by 10 and 50 redox cycles from 0.05 to 1.25 V_RHE_ at 50 mV/s in 0.1 M H_2_SO_4_. To showcase the dissolution of ruthenium oxides at anodic potentials, micrographs were recorded before and after polarizing the catalyst at 1.55 V_RHE_ for 5 min (chronoamperometry).

### Computational Methods

To interpret the experimental O K-edge spectra, we simulated O K-edge spectra for oxygen species on rutile RuO_2_ surfaces and extended them to include bulk anatase and hollandite RuO_2_. We employed the Quantum ESPRESSO package version 6.1.^46^ Ground-state calculations were performed using the PBE functional^47^ with projector augmented wave data sets taken from the PS Library^48^ and a kinetic energy (charge density) cutoff of 60 Ry (600 Ry). The surface models contained at least five layers of RuO_2_, of which all except the central two layers were allowed to relax. A *k*-point mesh equivalent to at least (8 × 8) for the (1 × 1) (001) rutile surface unit cell was used in conjunction with cold smearing^49^ (smearing parameter 0.02 Ry). To compute the O K-edge spectra, we used a one-electron Fermi’s golden-rule expression implemented in XSpectra.^50,51^ We employed the same *k*-point mesh as that used for the ground-state calculations and did not make use of a core hole potential. ΔSCF calculations were used to determine the relative edge positions. Subsequently, the absolute excitation energy scale of the computed spectra was set using the white-line energy of bulk rutile RuO_2_, which is located at 529.6 eV. The calculations on the anatase and hollandite structures were performed on a bulk unit cell. To generate μ_2_-O species within this unit cell, an Ru vacancy was introduced.

## Results and Discussion

To characterize the electrochemical properties of the amorphous and crystalline RuO_*x*_ films studied here, cyclic voltammograms (CVs) and linear sweep voltammograms (LSVs) were obtained in an RDE setup. For both oxides, broad redox features are observed between 0.6 and 0.8 V_RHE_ during the potential cycling, which is characteristic for ruthenium oxides. These features are generally attributed to the Ru^3+^/Ru^4+^ redox couple.^42,52^ At lower potentials, clear differences are observed for the two oxides. For amorphous RuO_*x*_, low currents are observed as expected for amorphous RuO_*x*_ due to the lower conductivity of the oxide in its bulk 3+ state.^53,54^ In contrast, the crystalline RuO_2_ film shows pronounced redox features at around 0.2 V_RHE_, which are commonly observed for polycrystalline ruthenium oxides^55^ and are attributed to hydrogen adsorption at the undercoordinated ruthenium. Hence, the electrochemical characterization confirms the amorphous and crystalline nature of the two types of films employed here. Further evidence for this is provided using XAS in SI Section S5.

**Figure 2:**
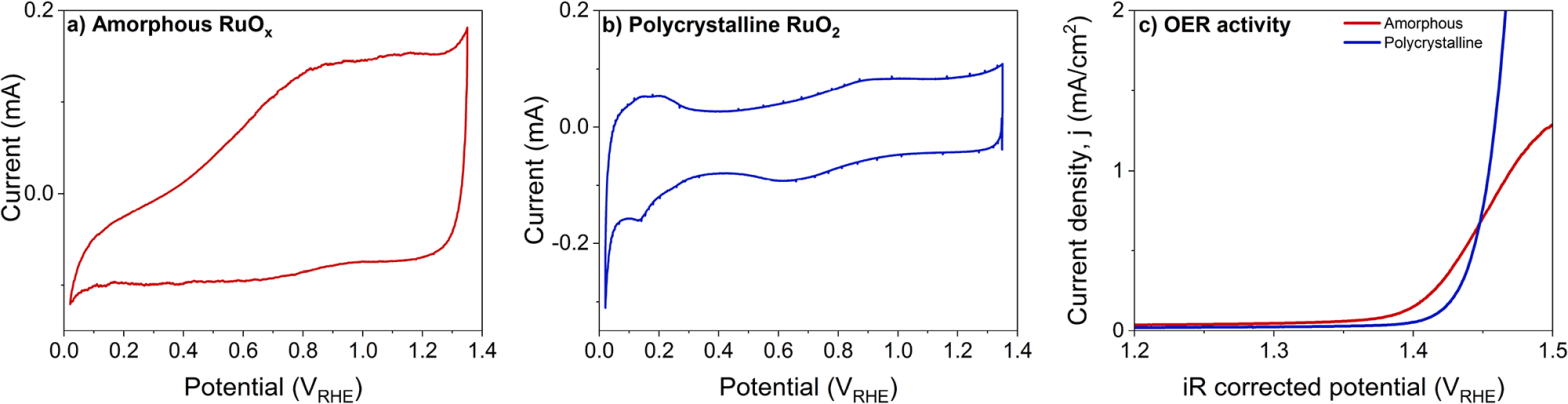
OER activity of ruthenium oxide films. (a) Cyclic voltammogram of amorphous ruthenium oxide recorded in the RDE setup using 0.1 M H_2_SO_4_ as electrolyte at 50 mV/s. (b) Cyclic voltammogram of polycrystalline ruthenium oxide recorded in the RDE setup using 0.1 M H_2_SO_4_ as electrolyte at 50 mV/s. (c) Linear sweep voltammetry (LSV) at 5 mV/s showing the OER activity of amorphous and polycrystalline ruthenium oxide in 0.1 M H_2_SO_4_ as electrolyte.

Using LSVs, we compared the OER activity of the two films. From Figure 2c, it is clear that the OER onset potential of the amorphous oxide is lower than its crystalline counterpart, in line with the higher activity expected for the amorphous oxide.^22,23^ The difference in electrocatalyst stability is also immediately apparent from the measurement. For amorphous RuO_*x*_, the dissolution rate under OER conditions is so high that significant deactivation occurs during the LSV sweep. As a result, only a low slope is observed, and the current even levels off at high overpotential. This was also visually observed in the electrode in the form of erosion of the catalyst layer after the measurement. Again, this is in line with the literature, confirming the trade-off between activity and stability often observed for amorphous and crystalline OER catalysts.^26,56^

**Figure 3:**
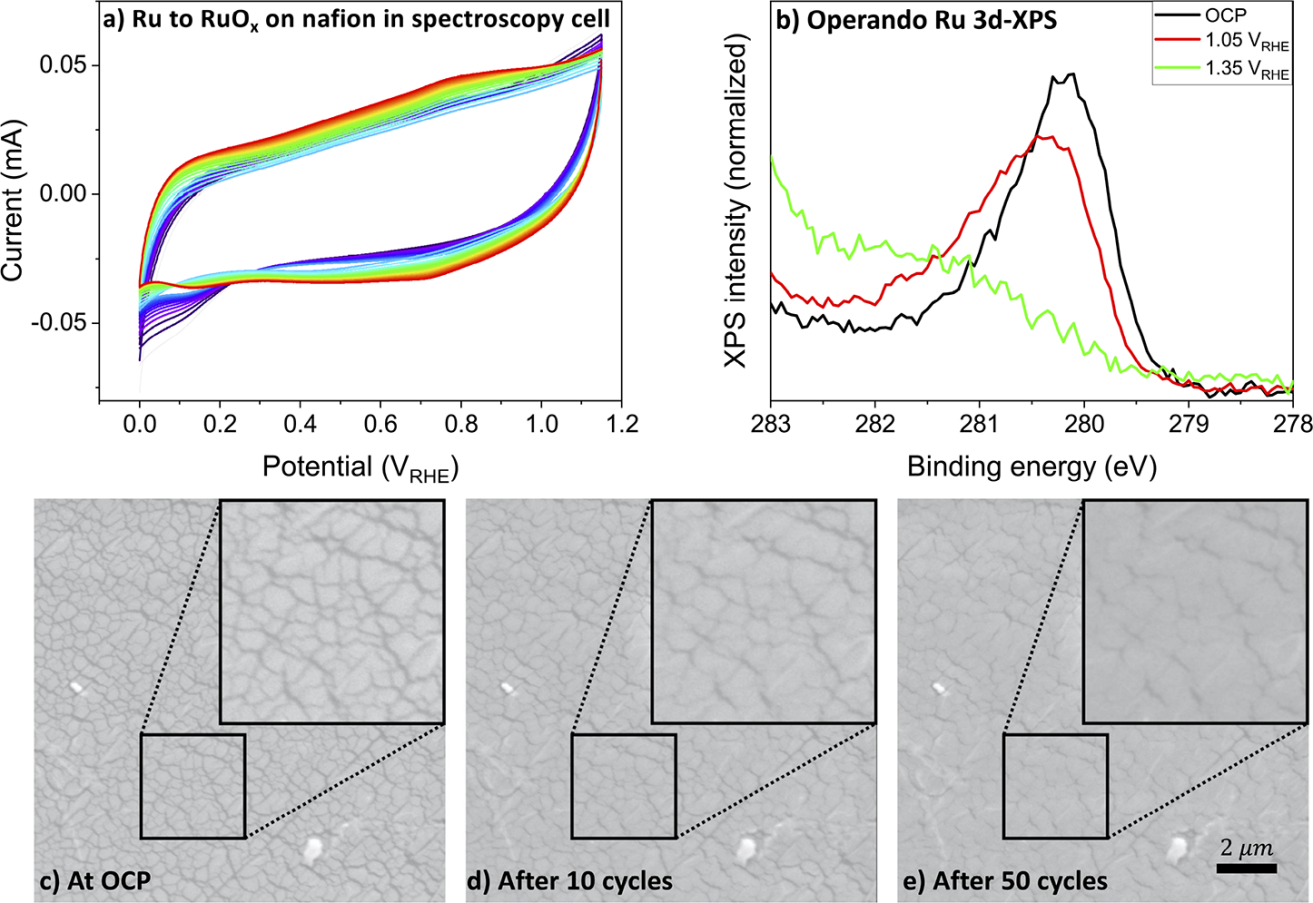
Electrochemical oxidation of sputter deposited ruthenium films. (a) Cyclic voltammogram of metallic ruthenium recorded in the spectroscopy cell using 0.1 M H_2_SO_4_ as electrolyte at 50 mV/s. The initial cycles are purple and later are red in color. (b) Operando Ru 3d XPS of a metallic ruthenium film sandwiched between graphene and Nafion membrane. (c–e) Operando electrochemical SEM micrographs of a ruthenium film covered with graphene at OCP, after 10 cycles, and after 50 cycles of anodic polarization.

To confirm the validity of our spectroelectrochemical approach, we first studied the oxidation of the sputter deposited Ru layer. The CVs (Figure 3a) show the electrochemical conversion of the sputter deposited metallic ruthenium film to RuO_*x*_ inside the IEM spectroscopy cell. The broad peak emerging at ∼0.8 V_RHE_ with the increase in number of cycles, corresponding to the Ru^3+^/Ru^4+^ redox couple, confirms an equivalent electrochemical response of the RDE setup and the operando cell used for XAS.

The conversion of the metallic film to oxide is confirmed by operando Ru 3d XPS (Figure 3b). At OCP, the Ru 3d_5/2_ peak occurs at a binding energy of 280.1 eV, which indicates the presence of metallic ruthenium.^57,58^ We should note that we only analyze the Ru 3d_5/2_ peak here because the Ru 3d_3/2_ overlaps with the C 1s peak of the graphene and Nafion in the cell. With increase in applied potential, the peak shifts to higher binding energy and broadens, which confirms the oxidation of ruthenium, at least within the probing depth of the measurement (a few nanometers). As expected for an electrochemically oxidized film, the broad shape of the Ru 3d_5/2_ peak at 1.35 V_RHE_ suggests that the oxide is an amorphous hydrous RuO_*x*_.

The oxidation of the Ru film can be visualized using EC-SEM. At open circuit, the sputter deposited ruthenium film shows a microstructured pattern (cracks) that results from the expansion of the underlying Nafion membrane upon contact with the electrolyte (Figure 3c). When the layer is oxidized during CV cycles (Figure 3d,e), the film expands and shows less intense cracks. This is due to the expansion of the lattice during conversion from metallic ruthenium to RuO_*x*_. The lattice expansion of the film is uniformly observed over the entire surface, confirming that the entire sample is electrochemically responsive in our confined electrolyte geometry. Hence, we may expect a uniform response to the applied potential in the XAS studies that follow.

### Oxygen K-Edge XAS

**Figure 4:**
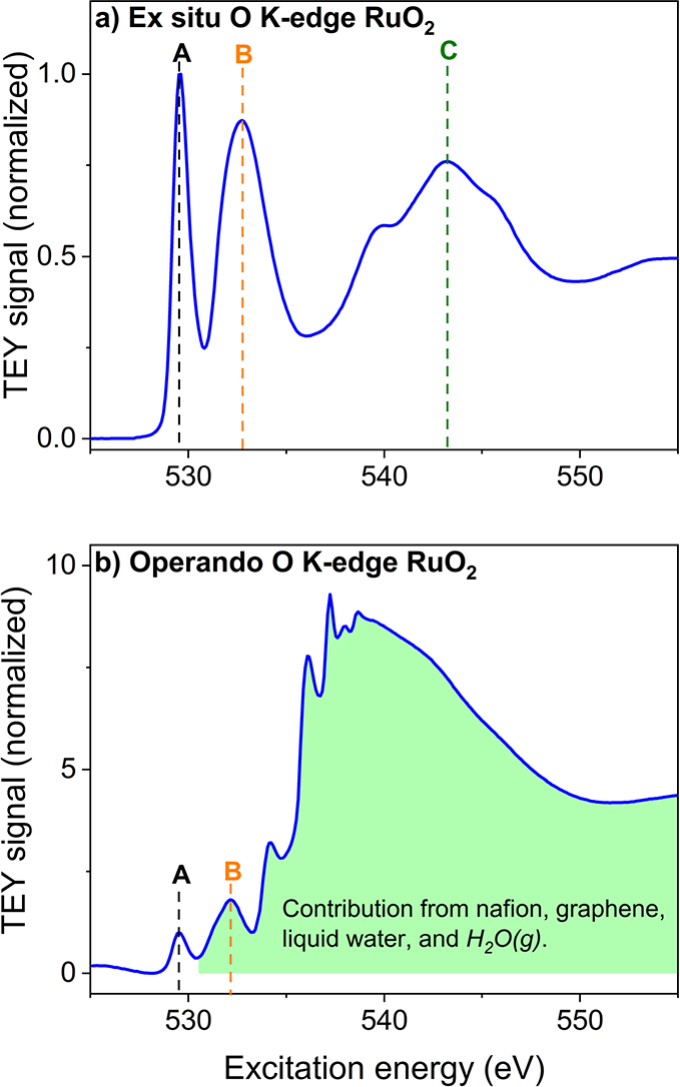
Comparison of ex situ and operando O K-edge XAS. (a) Ex situ O K-edge spectrum of polycrystalline RuO_2_ powder (Alfa Aesar) showing resonances at 529.6 eV (A, black dashed line), 532.7 eV (B, orange dashed line), and 542.8 eV (C, green dashed line). (b) operando O K-edge spectrum of polycrystalline RuO_2_ recorded in the IEM spectroscopy cell at 1.25 V_RHE_. Both the spectra are normalized to μ_3_-O intensity at 529.6 eV.

To probe the evolution of oxygen species in the RuO_*x*_ electrode at potentials from OCP up to the OER, we used operando O K-edge XAS. The O K-edge spectrum of ruthenium oxides shows several resonances that can be used to extract chemical information about the environment of the oxygen atoms in the material. We will first discuss these resonances using the ex situ spectrum of rutile RuO_2_. As shown in Figure 4a, the spectrum consists of two sharp peaks at 529.6 eV (A) and 532.7 eV (B). These can be attributed to electronic transitions from O 1s to unoccupied O 2p-Ru 4d_t_2g_ hybridized orbitals and O 1s to unoccupied O 2p-Ru 4d_e_g_ hybridized orbitals, respectively. At higher X-ray energies, a broader feature arises at 542.8 eV (C) corresponding to excitations from O 1s to hybridized O 2p-Ru 5sp hybridized orbitals.^59,60^ The operando spectrum of rutile RuO_2_ (Figure 4b) is different from the ex situ spectrum in the higher-energy regime beyond 531 eV because of contributions of the components of the sandwiched assembly (e.g., water, Nafion, and functional groups on graphene). An assignment of all resonances observed in the operando spectra is provided in SI Section S3.

For the analysis of the operando O K-edge spectra, we will focus on the lowest energy resonance (O 1s → O 2p-Ru 4d_t_2g_), the so-called white line. This resonance has excellent chemical sensitivity, as we will show in the following sections. In addition, note that the energy region between 525 and 531 eV does not contain resonances from other oxygen species in the cell (e.g., water, Nafion, functional groups on graphene), making the interpretation of the resonances in this energy region unambiguous.^35,36^

**Figure 5:**
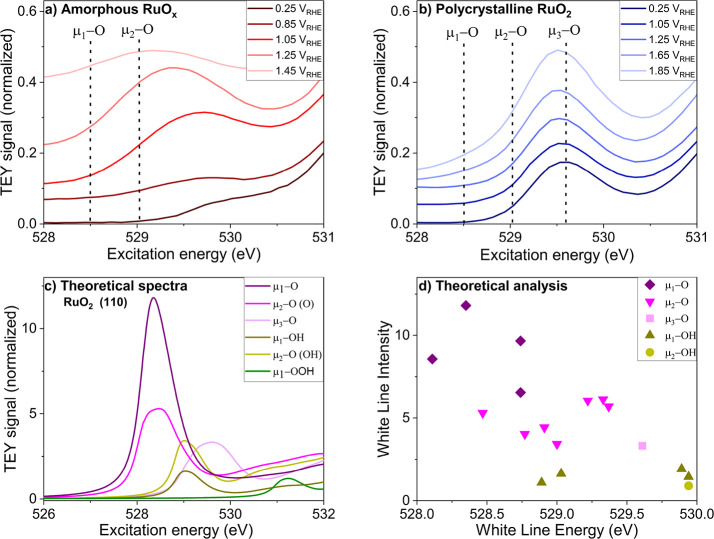
Operando O K-edge XAS of RuO_*x*_ films on Nafion with 0.1 M H_2_SO_4_ as electrolyte. (a, b) Experimental spectra of amorphous RuO_*x*_ and polycrystalline RuO_2_. Both the spectra are normalized to the edge jump at 551.15 eV and have been plotted with a *y*-offset for clarity. (c) Theoretical spectra calculated for various oxygen species on RuO_2_(110). (d) Theoretical white line energy vs intensity of various single crystalline facets (RuO_2_ (110), (100), (101)) (more details in SI Section S4).

Using the RuO_*x*_ white line in the operando O K-edge spectra, we have followed the structural evolution of the electrochemically oxidized ruthenium oxide as a function of the applied potential. As shown in Figure 5a, there is a pronounced increase in white line intensity when the potential is increased, accompanied by a shift of the peak to lower excitation energy. This indicates a significant change in the structure of the oxide. The behavior differs from the case of rutile RuO_2_ (Figure 5b), where the changes are much less pronounced. In this case, we only observe a small white line shift and increase in intensity at higher applied potential along with the rise of a shoulder at low excitation energy.

To interpret these observations in the experimental data, O K-edge spectra were simulated for a range of oxygen species on various single crystalline ruthenium oxide surfaces using DFT. A comparison between the experimental and theoretical white line peaks allows us to distinguish different oxygen coordination environments and tell us whether -O or -OH is present. We use the μ-nomenclature to describe these different kinds of oxygen species, in which μ*_1_*, μ*_2_*, and μ*_3_* designate oxygen atoms bound to 1, 2, or 3 Ru atoms, respectively (see Figure 1 for a ball and stick model). As displayed in Figure 5c,d, the simulations show that the energy and intensity of the different oxygen species are strongly dependent on the coordination number and protonation state. For example, on a RuO_2_ (110) surface (Figure 5c), the μ*_1_*-OH white line (dark greenish yellow color) occurs at 529 eV, whereas its deprotonated form (μ*_1_*-O) results in a white line (dark purple color) with higher intensity at a lower excitation energy of 528.3 eV. The intensity and position of the peak for bridging oxygen sites (μ*_2_*-O) vary according to the protonation status of its neighbor (μ*_1_*-O or μ*_1_*-OH) oxygen species situated on top of an Ru_cus_ site. Theoretical calculations for other surfaces have been described in SI Section S4. The collective information from the theoretical calculations on various RuO_2_ surfaces is summarized in Figure 5d, where it can be seen that the deprotonated (-O) species have higher resonance intensity per atom and a white line at lower excitation energy compared to the corresponding protonated (-OH) species. Hence, two clear guidelines can be formulated from the DFT calculations: first, a rise in intensity of the peak in the operando experimental spectra would suggest conversion of -OH species to -O species (deprotonation); second, on the basis of the approximate positions of the white line, the rising peaks could be assigned to a specific oxygen species. Although the white line peak positions in Figure 5d show some scatter, we can assign the following positions: μ*_1_*-O → 528.5 eV, μ*_2_*-O → 529 eV, and μ*_3_*-O → 529.6 eV. Thus, using the white line peak positions and intensity observed in the operando spectra, we can visualize the changes in the oxygen species present in the oxides at various applied potentials.

Using the guidelines obtained from our theoretical analysis, it is clear that the oxides become increasingly deprotonated when the potential is increased. In the amorphous RuO_*x*_ sample (Figure 5a), the weak shoulder at approximately 530 eV observed at 0.25 and 0.85 V_RHE_ indicates that the sample is strongly hydroxylated both in the bulk and at the surface. This is expected, as an electrochemically oxidized ruthenium oxide is hydrous (RuO_*x*_·*x*H_2_O) in nature.^27^ Thus, at low applied potential, both the surface and subsurface regions of an amorphous oxide predominantly contain μ*_1_*-OH, μ*_1_*-OH_2_, and μ*_2_*-OH and potentially a small fraction of μ*_3_*-O species.

With the increase in applied potential (1.05 and 1.25 V_RHE_), the white line intensity rises and shifts to approximately 529.3 eV, indicating the formation of μ*_2_*-O species analogous to recent observations on IrO_*x*_.^36^
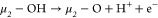
1

**Figure 6:**
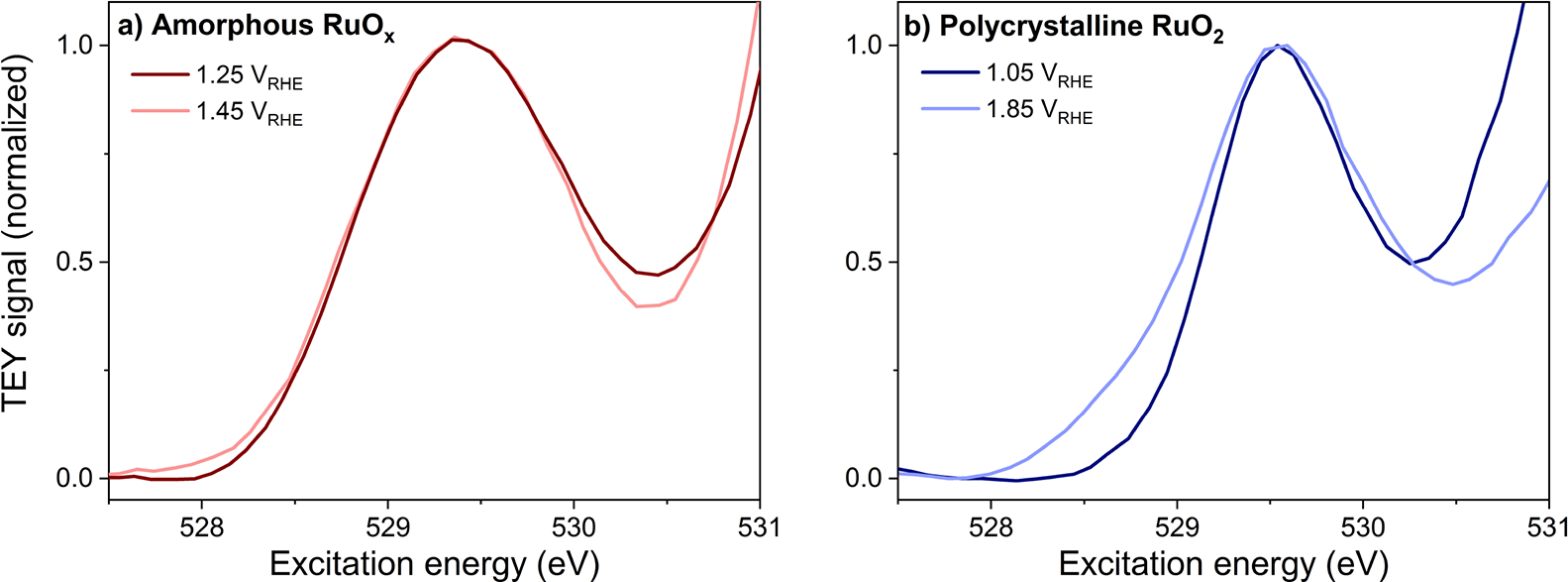
Comparison of O K-edge white line shape. (a) O K-edge spectra of amorphous ruthenium oxide at 1.25 and 1.45 V_RHE_ where the 1.45 V_RHE_ white line is shifted by 0.2 eV toward higher excitation energy to overlay. (b) O K-edge spectra of polycrystalline ruthenium oxide at 1.05 and 1.85 V_RHE_ where the 1.85 V_RHE_ white line is shifted by 0.04 eV toward higher excitation energy to overlay. All the peaks are normalized to the peak intensity at 529.6 eV.

At 1.45 V_RHE_, the peak further shifts to lower excitation energy. This shows that the deprotonation of μ*_2_*-OH species continues at higher potentials. This is in accordance with the theoretical prediction that the peak position of μ*_2_*-O species shifts to lower excitation energy when its neighbor oxygen species are deprotonated (greenish yellow vs purple color in Figure 5c): as more and more μ*_2_*-OH is converted to μ*_2_*-O, there will be an increasing number of neighboring μ*_2_*-O sites, resulting in a peak shift. As illustrated in SI Section S7, the oxides revert back to the protonated state when the applied potential is lowered back to 0.25 V_RHE._ A closer comparison of the white line at 1.25 and 1.45 V_RHE_ in Figure 6a (normalized and overlaid) shows that the white line at 1.45 V_RHE_ slightly broadens at around 528 eV. This could occur because of the rise of intensity at 528.5 eV, implying the formation of μ*_1_*-O species at OER relevant potentials.
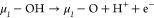
2

**Figure 7:**
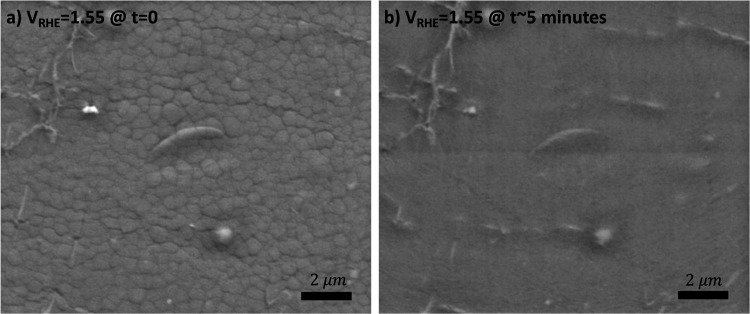
Dissolution of ruthenium oxide at anodic potentials. (a) EC-SEM image at 1.55 V_RHE_ initially displaying the microstructured cracking pattern of the RuO_*x*_ film. (b) EC-SEM image at 1.55 V_RHE_ after approximately 5 min showing that the RuO_*x*_ film has dissolved, leaving a smooth Nafion surface behind. Note that some wrinkles in the graphene window are also visible in the images.

However, it should be noted that the shoulder is very weak, which suggests that most of the μ*_1_*-sites do not contain μ*_1_*-O. Again, this is similar to recent observations on IrO_*x*_,^36^ showing the similarity in the behavior of these OER-active oxides. Note in Figure 5a that the total intensity of the white line has dropped at 1.45 V_RHE_ as a result of catalyst dissolution. Indeed, the white line completely disappeared within roughly 10 min, indicating the complete dissolution of the film. This is confirmed by EC-SEM images in Figure 7a,b, which show that the film has completely dissolved into the Nafion membrane at OER potentials. This is in line with literature observations, which indicate that amorphous ruthenium oxide is highly unstable under OER conditions.^14,24,25^ The dissolution is thought to proceed via RuO_4_ species.^61,62^ One may hypothesize that these contribute to the operando O K-edge spectrum at 1.45 V_RHE_. However, as we will show in the analysis of Ru M-edge spectra, the concentration of high oxidation state species such as RuO_4_ is below the detection limit, precluding a significant contribution to the O K-edge.

For crystalline RuO_2_, the O K-edge spectra can be interpreted in a similar fashion as for the amorphous RuO_*x*_. At an applied potential of 0.25 V_RHE_, the rutile RuO_2_ sample (Figure 5b) exhibits a sharp peak at approximately 529.6 eV that can be attributed to μ*_3_*-O oxygen species. This is the only oxygen species present in the bulk of crystalline RuO_2_, and hence, it can indeed be expected to dominate the signal.^27^ The absence of any shoulder or broadening toward lower excitation energies suggests that the surface is protonated at 0.25 V_RHE_. Indeed, at such low applied potential, we may expect the surface to be occupied by μ*_1_*-OH or μ*_1_*-H_2_O groups and μ*_2_*-OH groups. Increasing the potential results in a broadening of the white line toward lower energy, along with an increase in intensity. This can be explained by the emergence of a μ*_2_*-O white line at ∼529.2 eV via reaction 1, in line with the observations for amorphous RuO_*x*_. Because the broadening and intensity increase proceed over the entire potential range probed here, we can conclude that the transition from μ*_2_*-OH to μ*_2_*-O proceeds gradually, again similar to the observation for amorphous RuO_*x*_.

Although the amorphous RuO_*x*_ and crystalline RuO_2_ thus show qualitatively similar behavior, they clearly differ in the degree to which the μ*_2_*-O peak shifts as more and more μ*_2_*-O is formed. Comparing Figure 5a,b, we see that the peak shift is larger in the amorphous oxide as compared to its crystalline counterpart. This signifies that the effect of neighbor interactions is strong in the amorphous oxide, whereas it is quite modest in the crystalline oxide. This can be explained based on the structure of the oxides: The amorphous oxide contains μ*_2_*-O(H) groups both at the surface and in the bulk. Hence, when μ*_2_*-OH groups are oxidized, this leads to a large change in the electronic structure throughout the entire oxygen lattice. In contrast, in crystalline oxide, the μ*_2_*-O(H) groups are only located at the surface. As a result, there is only a mild change in the electronic structure of the oxygen lattice. In other words, the lattice gets more “activated” as a result of deprotonation events in amorphous oxides as compared to their crystalline counterparts. This notion fits with the observation that the oxygen lattice participates in the OER for amorphous RuO_*x*_,^63^ whereas no lattice oxygen involvement was observed for crystalline RuO_2_.^64^ This difference in oxygen lattice activation may explain both the higher activity and lower stability of amorphous RuO_*x*_, as we will discuss in more depth in the next section.

Because the rutile RuO_2_ sample is fairly stable under OER conditions, we were able to measure deep into the OER regime (Figures 5b and 6b). At such high potentials (1.65 and 1.85 V_RHE_), a small shoulder appears at around 528.5 eV, which is likely due to the formation of μ*_1_*-O species (reaction 2). μ*_1_*-O is the oxygen species that is coordinated to a single Ru_cus_ site and is a reaction intermediate in most of the OER reaction mechanisms proposed in the literature.^2,9,65^ However, only a small μ*_1_*-O signal is observed for RuO_2_ despite the high white line intensity of this species (dark purple color in Figure 5c). Similar to the case of amorphous RuO_*x*_, this means that the μ*_1_*-O reaction intermediate does not dominate the surface. In line with this, Divanis et al.^66^ proposed an alternative structure for the μ*_1_*-sites just below the OER onset, consisting of a μ*_1_*-O---H—O-μ*_2_* complex in which a proton is shared between the μ*_1_*-O and μ*_2_*-O species. Intuitively, this may result in a white line similar to a protonated species with less intensity and at an excitation energy higher than 528.5 eV, consistent with our observations. At potentials above the OER onset, Rao et al.^67^ reported that the μ*_1_*-sites are occupied by μ*_1_*-OOH species, also in line with a low μ*_1_*-O signal in our spectra. Our theoretical calculations show that μ*_1_*-OOH results in an O K-edge resonance around 531 eV (green color in Figure 5c), similar to molecular O_2_. This region cannot be quantitatively analyzed in our experimental spectra because of the overlap with C–O signals from the Nafion membrane.

### Ruthenium M_3_-Edge XAS

**Figure 8:**
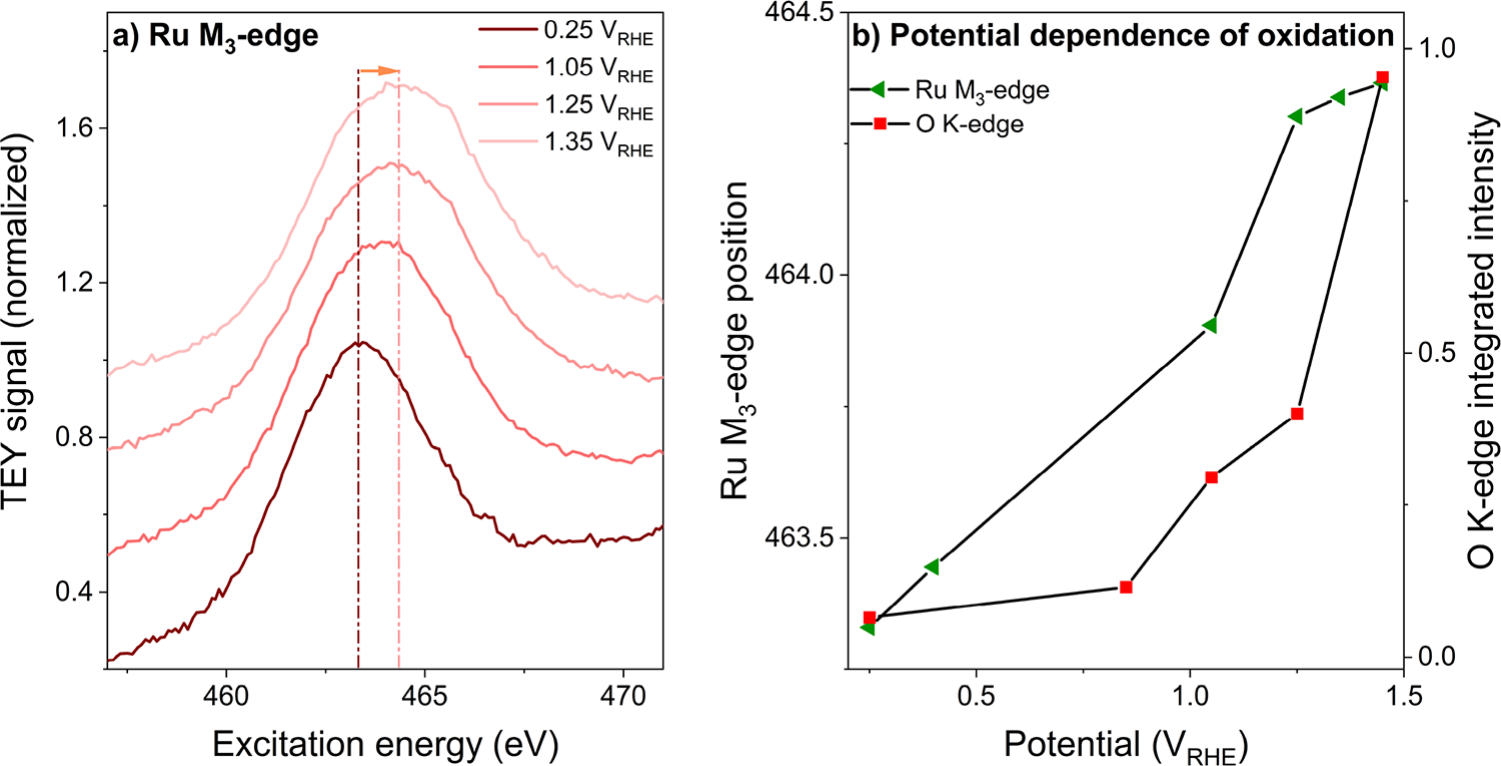
Potential dependence of Ru oxidation and OH deprotonation. (a) Operando Ru M_3_-edge spectra of RuO_*x*_ films on Nafion using 0.1 M H_2_SO_4_ as electrolyte. (b) Comparison between Ru M_3_-edge peak position (marker of Ru oxidation state) and O K-edge intensity (marker for OH deprotonation level). The O K-edge intensity was obtained by integration of the O K-edge spectra of amorphous RuO_*x*_ between 528 and 530 eV.

So far, we have seen that the increase in applied potential leads to oxidative deprotonation of the RuO_*x*_ catalyst, i.e., the conversion of -OH groups to -O or -OOH. This process may also affect the oxidation state of the ruthenium atoms. To probe the changes in the electronic structure of ruthenium, Ru M_3_-edge spectra of the electrochemically oxidized RuO_*x*_ film were recorded in the same experiment as the O K-edge spectra. The operando spectra in Figure 8a show that the Ru M_3_ white line peak shifts to higher excitation energy with the increase in applied potential, indicating an increase in Ru oxidation state. At 0.25 V_RHE_, the M_3_ white line peak occurs at 463.3 eV, whereas a shift to 464.3 eV is observed at 1.35 V_RHE_. Comparing this to the Ru M_3_ peak positions of Ru^0^ (462.19 eV) and Ru^4+^ (463.96 eV) obtained from reference spectra (figure in SI Section S8), the operando spectra hint that the average oxidation state of ruthenium reaches Ru^4+^ at the onset of OER, possibly with a small fraction of Ru^5+^. This trend was confirmed by Ru L_3,2_-edge spectra on an amorphous RuO_*x*_ powder, which permit a more precise quantitative analysis based on the white line intensity (SI Section S9). The trend in Ru redox is strongly reminiscent of the electrochemical oxidation of IrO_*x*_, where the transition from Ir^3+^ to Ir^4+/5+^ was observed.^33^ Again, this underlines the similarity between these catalysts.

By comparing the trend in Ru M_3_-edge position to the trend of the intensity of the O K-edge white line, we can decouple the oxidation events occurring at the ruthenium and oxygen sites at various applied potentials. As shown in Figure 8b, the Ru and O atoms follow a very different oxidation trend as the potential is increased. Oxidation of the Ru atoms is already observed at 0.35 V_RHE_, whereas the onset of μ*_2_*-OH deprotonation is not observed until 0.85 V_RHE_. In analogy to the case of IrO_*x*_,^36^ we can explain these observations by a third surface reaction:
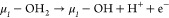
3

All species involved in this reaction have a very low O K-edge white line intensity and can therefore not readily be observed. However, the reaction does oxidize the electrode. On the basis of the Ru M_3_-edge position, it appears that this oxidation is ruthenium-centered. Beyond 1.0 V_RHE_, the situation is reversed: the Ru M_3_-edge position remains almost constant, whereas the O K-edge intensity is dramatically increasing. As noted before, the increase in O K-edge intensity is mainly caused by the formation of μ*_2_*-O via reaction 1. Again, this is an oxidative process. Because the Ru M_3_-edge position remains almost constant during this oxidative reaction, we conclude that the oxidation is oxygen-centered.

The observations from Figure 8 provide a clear picture of how RuO_*x*_ is activated for the OER. First, we see that the extent of oxidation on the Ru atoms is limited. Rather, it seems like the oxygen-centered oxidation that occurs around the onset potential of the OER is essential for driving the catalysis. Analogous to the case of IrO_*x*,_^36^ we can interpret this by considering that oxygen-centered oxidation results in electrophilic oxygen species, which are reactive toward the O–O coupling that is crucial for the OER. For amorphous RuO_*x*_, the activation of the entire lattice appears to be very pronounced based on the significant shift of the μ*_2_*-O white line. Combining this with the observation in the literature that lattice O atoms participate in the OER in amorphous ruthenium oxide,^63^ we conclude that the oxygen-centered oxidation in this material creates sufficient electrophilic character in the lattice (μ*_2_*-)O atoms to participate in the O–O coupling step of the OER. Unfortunately, the lattice activation also appears to lead to increased RuO_*x*_ dissolution. Little is known about the mechanism of the dissolution process, but we speculate that more electrophilic lattice O atoms more readily undergo Ru–O bond scission. Thus, oxygen-centered oxidation seems to be a double-edged sword affecting both activity and stability.

### Conclusions

Using operando XAS, we have probed the redox events occurring at the Ru and O atoms in ruthenium oxides at potentials from open circuit to the oxygen evolution reaction range. Our data show that the material is strongly hydrated at low potentials but becomes increasingly deprotonated as the potential approaches the onset of the OER range. The Ru and O atoms in the lattice are both significantly impacted by this deprotonation. Initially, it is primarily the Ru atoms that are oxidized, saturating at Ru^4+^, possibly with a small fraction of Ru^5+^. However, at potentials above ∼1.2 V_RHE_, it is the oxygen lattice that is oxidized. According to our O K-edge spectra, the entire oxygen lattice is affected both at the surface and in the bulk of the material. This activation of the oxygen lattice is much more pronounced for the amorphous RuO_*x*_ than for polycrystalline RuO_2_. We propose that this accounts for the higher activity and lower stability of amorphous RuO_*x*_ as compared to the crystalline RuO_2_. Overall, this study highlights the importance of oxidative deprotonation in acidic OER catalysts and shows how the oxygen lattice as a whole can be activated for the water splitting process but likely also for catalyst degradation. Thus, knowledge about the redox nature of oxygen atoms can be used as a tool for the design of future catalysts by obtaining an optimal balance between oxygen-centered and metal-centered oxidation. For pure ruthenium oxides, this balance appears to lie too far toward oxygen-centered oxidation, leading to low stability. Therefore, we envisage that designers of future ruthenium-based OER catalysts could aim in particular at using either cation dopants that can readily go beyond the 4+ oxidation state to favor metal-centered oxidation or anion dopants that inject negative charge into the oxygen lattice.

## Supporting Information Available

Details on the sample preparation and characterization, cell configuration, analysis procedures, and additional experimental and theoretical data (PDF)
