# Supplementary material for: On the Operando Structure of Ruthenium Oxides during the Oxygen Evolution Reaction in Acidic Media
Source: ACS Catal. 2023 May 19;13(11):7488–98. doi: 10.1021/acscatal.3c01607 (PMC10242682; doi:10.1021/acscatal.3c01607)
Supplement: Supplementary file 1 — cs3c01607_si_001.pdf [file cs3c01607_si_001.pdf]

Supporting Information

On the operando structure of ruthenium oxides during the oxygen evolution reaction in acidic media.

Nipon Deka[a], Travis E. Jones[b], Lorenz J. Falling[c], Luis-Ernesto Sandoval-Diaz[d], Thomas Lunkenbein[d], Juan-Jesus Velasco-Velez[d], Ting-Shan Chan[e], Cheng-Hao Chuang[f], Axel Knop-Gericke[d], Rik V. Mom*[a]*

[a] Leiden Institute of Chemistry, Leiden University, 2300 RA Leiden, The Netherlands.

[b] Theoretical Divison, Los Alamos National Laboratory, Los Alamos, NM, USA.

[c] Lawrence Berkeley National Laboratory, 1 Cyclotron Rd, Berkeley, CA 94720, USA

[d] Fritz Haber Institute of the Max Planck Society, Faradayweg 4-6, 14195 Berlin, Germany.

[e] National Synchrotron Radiation Research Center (NSRRC), Hsinchu, 30076, Taiwan.

[f] Department of Physics, Tamkang University, No. 151, Yingzhuan Rd, New Taipei city 25137, Taiwan

Corresponding author: n.deka@lic.leidenuniv.nl

S1 Operando SiN_x cell assembly

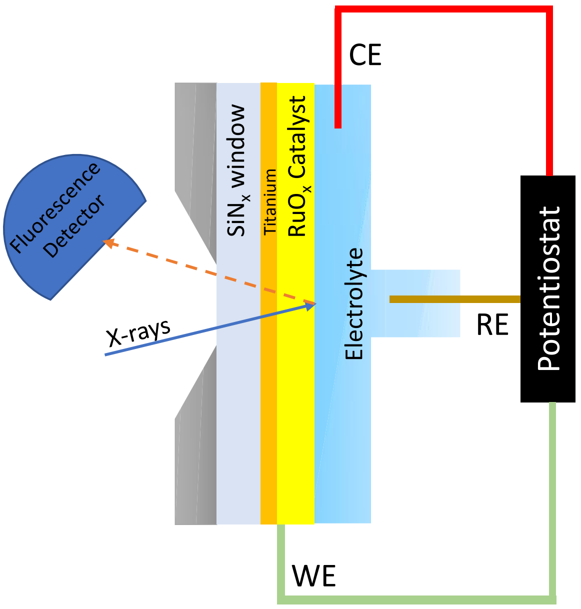

Figure S1: SiN_x flow cell assembly.

Commercially available hydrous $RuO_2.xH_2O$ powder used to record the Ru $L_{3,2}$-edge spectra in the SiN_x cell were purchased from Alfa Aesar. This compound is very similar to electrochemically oxidized RuO_xH_y. The hydrous powder was grounded in a mortar and dispersed in water to prepare an ink. The ink was drop casted on a SiN_x membrane coated with 20 nm Ti, and dried on a hot plate at 50 °C. A schematic representation of the SiN_x cell is shown in figure S1.

The SiN_x cell approach used for Ru $L_{3,2}$-edge XAS employed Pt wires as counter electrode and quasi-reference electrode in a three-electrode operando flow cell (see figure S1). The potential scale of the quasi-reference electrode was converted to the RHE scale using the OER onset potential. A SiN_x membrane supplied by Silson and coated with 20 nm Ti was used to support the RuO_x powder working electrode.

In the IEM cell used for the soft X-ray measurements, the reference electrode used was a DRIREF-2SH Ag/AgCl electrode. For easy comparison the potentials were later converted to the RHE scale. The electrical contact to the graphene/working electrode was made by a boron doped diamond coated niobium plate, which shows minimal background currents in the investigated potential range.

S2 Synthesis of polycrystalline ruthenium oxide

The polycrystalline RuO_2 films used in the IEM cell were prepared via calcination of sputter deposited ruthenium (figure S2). Firstly, Na_2SO_4 crystals were prepared by melting commercially available Na_2SO_4 powder (Sigma Aldrich, 99.99 %) in alumina boats at 905 °C for 2 hours. These crystals were broken into smaller pieces of approximately 5x5 mm. Metallic ruthenium was sputtered onto the Na_2SO_4 crystals (step 1 in figure S2) for 210 seconds in 0.1 mbar argon atmosphere with a sputter current of 40 mA which resulted in a film thickness of approximately 10-20 nm. Subsequent calcination (step 2) of the layer at 400 °C for 2 hours was used to crystallize the ruthenium oxide. After cooling, formvar solution (Sigma Aldrich) was drop casted (step 3) onto the RuO_2 layer for stabilization. In order to transfer the rutile RuO_2 layer to a nafion 117 proton exchange membrane, the whole assembly was gently suspended in pure water which dissolves the Na_2SO_4 substrate (step 4) leaving the RuO_2-formvar layer floating. Nafion 117 membranes purchased from Sigma Aldrich were cut into circular discs of approximately 11 mm diameter. These nafion discs were cleaned in an aqueous solution of 3% H_2O_2 at 80 °C for 2 hours followed by activation in 0.1M H_2SO_4 for another two hours at 80 °C. After cleaning, the discs were washed with milliQ water, dried in ambient air and stored for further use. Before use, nafion discs were briefly soaked in milliQ water to have a uniform wetting throughout. The RuO_2-formvar layer was scooped onto a clean nafion substrate (step 5). After drying for approximately 1 hour, the formvar layer was removed (step 6) using baths

of chloroform, acetone, ethanol and water. The RuO_2-on-nafion layer was covered with a bilayer of graphene (Graphenea) via scooping (step 8). Bilayer graphene on copper foil synthesized via CVD was purchased from Graphenea. This foil was suspended on a 40 g/L $(NH_4)_2S_2O_8$ etching solution with copper facing the solution side for 12-15 hours. This treatment etches away the copper leaving behind a free-floating graphene layer on the etching solution (step 7). The etching solution was replaced by milliQ water using a peristaltic pump. The RuO_2-on-nafion layer was gently introduced in the water to scoop the graphene on top of it (step 8). This assembly where the polycrystalline catalyst is sandwiched between a proton exchange membrane and a bilayer graphene was loaded onto an operando electrochemical cell for O K-edge and Ru M_3-edge XAS measurements.

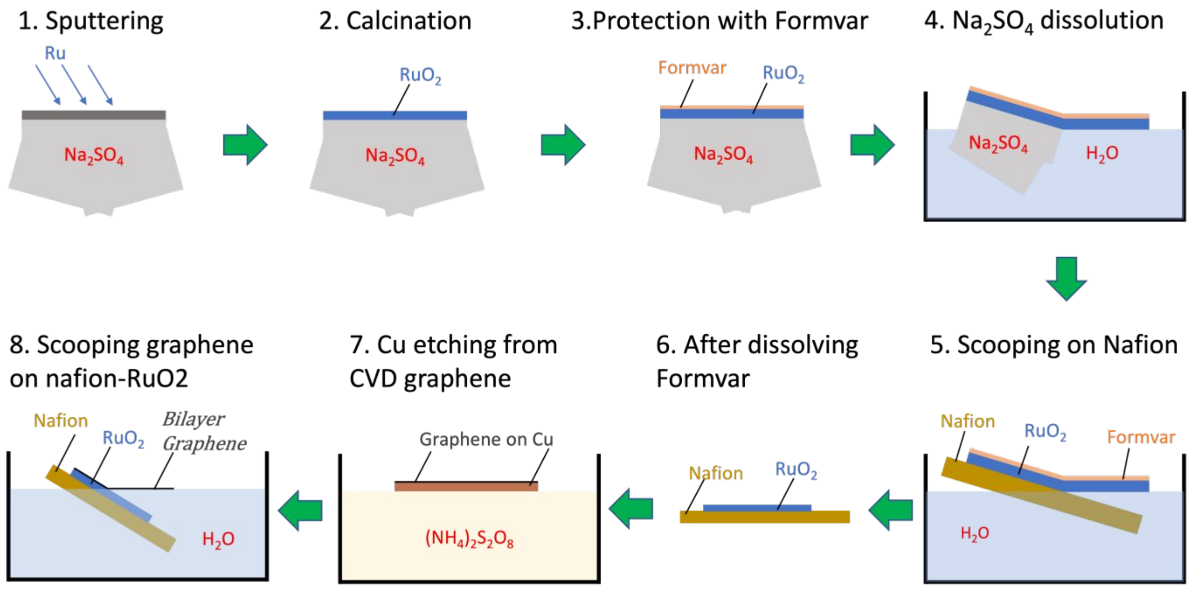

Figure S2: Synthesis and assembly of polycrystalline ruthenium oxide into confined electrolyte geometry.

S3 O K-edge XAS resonances

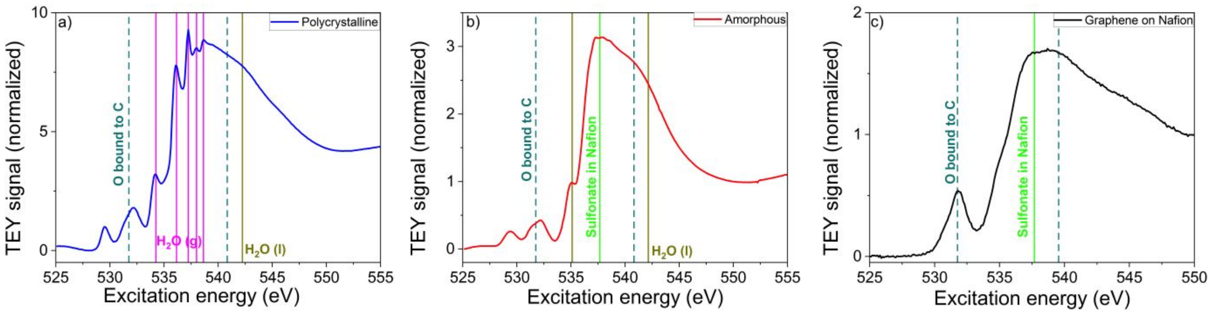

Figure S3: O K-edge XAS resonances identified using literature. *a) Operando O K-edge spectrum of polycrystalline ruthenium oxide at 1.25* V_{RHE} *showing resonances arising from water vapour (*$H_2O(g)$*) at 534.3 eV, 536.15 eV, 537.21 eV, 538 eV, and 538.62 eV, from liquid water (*$H_2O(l)$*) at 542.26 eV, nafion (O-C-O bond) at 531.77 eV and 540.87 eV. b) Operando O K-edge spectrum of amorphous ruthenium oxide at 1.25* V_{RHE} *showing resonances arising from liquid water (*$H_2O(l)$*) at 535.12 eV and 542.12 eV, from nafion (O-C-O) at 531.77 eV, and 540.76, from nafion (sulfonate group) at 537.67 eV. The spectra for the amorphous sample are normalized to the edge jump at 551.15 eV whereas the spectra for polycrystalline sample are normalized to* μ_3*-O intensity at 529.6 eV. c) O K-edge spectrum of dry graphene-covered nafion without catalyst (in the absence of electrolyte). The spectrum is normalized to the edge jump at 551.15 eV.*

In the confined electrolyte approach (IEM cell) used for operando O K-edge XAS, RuO_x is sandwiched between a nafion membrane and a bilayer graphene sheet. All the components involved in the sandwiched assembly and the water molecules in the electrolyte contain oxygen atoms and hence contribute to the O K-edge spectrum. The contribution of water in both liquid and gaseous phases can be seen in the operando spectrum (figure S3 a-b) of the RuO_x. For the gaseous phase of water ($H_2O(g)$), the peak at 534.3eV and 536.15 eV arises due to electronic transition in free water molecules from O 1s core orbital to 4a1 and 2b2 molecular orbitals

respectively[1]. The series of three closely spaced sharp peaks at 537.21eV, 538eV, and 538.62eV arises due to excitation of O 1s core electrons into Rydberg orbitals composed of hybridized 3p and 4p orbitals. The liquid phase of water ($H_2O(l)$) due to its condensed nature results in a O K-edge spectrum quite different from gaseous phase. The condensed phase of water shows contribution of hydrogen bonds in the spectrum[2,3]. The pre-peak at 535 eV results from broken/dangling hydrogen bonds, main edge at approximately 537 eV corresponds to population of molecules with unsaturated hydrogen bonds, and post edge at around 540 eV denotes the formed/strong hydrogen bonds which is enhanced in ice-like tetrahedral coordination[4]. In order to analyze the contribution of graphene and nafion, we have recorded the O K-edge spectrum of dry graphene-covered nafion (not wetted by electrolyte) (figure S3-c). The ether group in nafion results in a sharp peak at 531.75 eV and a broad feature at approximately 540.5 eV[5]. The sulfonate group in nafion results in a feature at approximately 537.6 eV.

S4 DFT calculation (all surfaces)

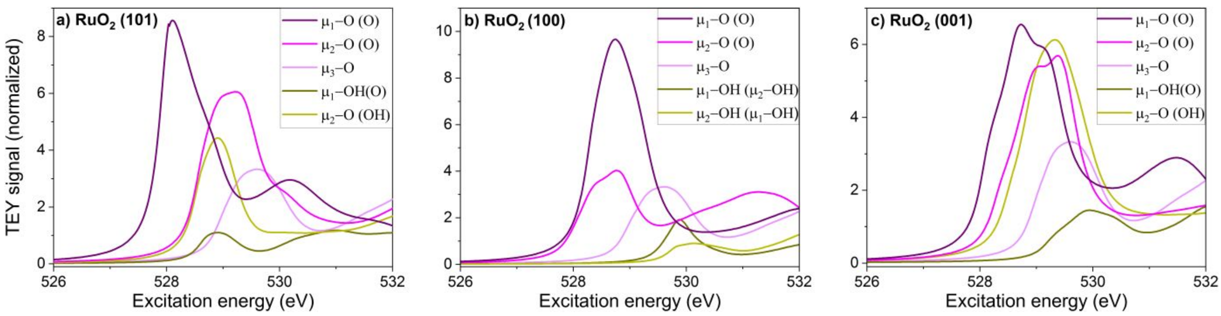

Figure S4: Theoretically calculated spectra showing the O K-edge resonances of various oxygen species on single crystalline RuO_2 surfaces. a) RuO_2 (101), b) RuO_2 (100), c) RuO_2 (001).

S5 Crystallinity of ruthenium oxide based on O K-edge XAS

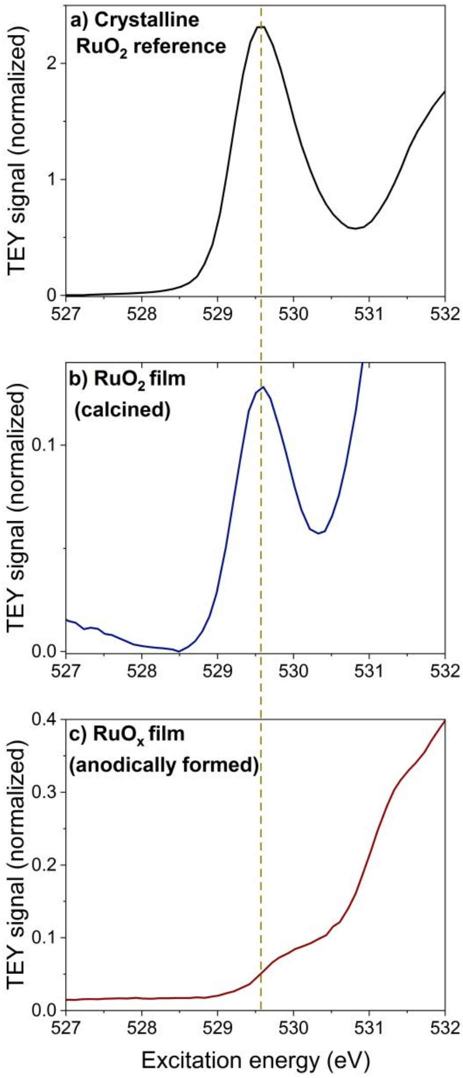

Figure S5: Crystallinity from O K-edge XAS. a) O K-edge spectrum of crystalline RuO_2 powder (Alfa Aesar) showing resonance at 529.6 eV arising from μ_3-O oxygen species. b) O K-edge spectrum of ruthenium oxide synthesized by calcination of metallic ruthenium at 400 °C polarized at 0.25 V_{RHE}. c) O K-edge spectrum of ruthenium oxide synthesized electrochemically by potential cycling of sputter deposited metallic ruthenium polarized at 0.25 V_{RHE}. All the spectra are normalized to edge jump at 551.15 eV.

O K-edge spectra can be used to determine the crystallinity of the synthesized ruthenium oxide films. The unit cell of crystalline ruthenium oxides contains only μ_3-O oxygen species[6,7].

Hence, the μ_3-O feature at 529.6 eV can be used as an indicator for the level of crystallinity of the films, analogous to previous work on iridium oxides[7]. A relatively intense signal at 529.6 eV signifies that the sub-surface and bulk region of the oxide contains a majority of μ_3-O species. This is the situation in the case of reference polycrystalline RuO_2 sample bought from Alfa Aesar and the RuO_2 films calcined at 400 °C as shown in figure S5 (a) and (b) respectively, confirming the crystallinity of the calcined RuO_2 film used here. In contrast, the electrochemically synthesized film shows a very weak signal at 529.6 eV. This indicates that the sub-surface region mostly contains hydrous groups such as μ_1-OH, μ_1-OH_2, and μ_2-OH. This is consistent with an amorphous RuO_x film, where one can expect mainly undercoordinated μ_1 and μ_2 oxygen sites, which become protonated in aqueous conditions.

S6 Shift of O K-edge XAS resonances and effect of normalization

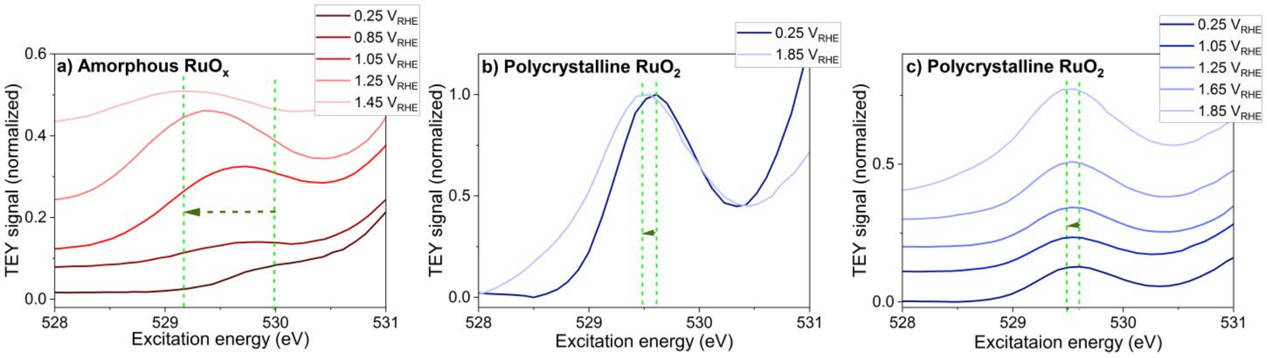

Figure S6: Operando O K-edge XAS of RuO_x films on Nafion with 0.1M H_2SO_4 as electrolyte. *a) Experimental spectra of amorphous RuO_x normalized to the edge jump at 551.15 eV and plotted with a y-offset. b) Experimental spectra of polycrystalline RuO_2 normalized to the μ_3-O intensity at 529.6 eV and plotted on top of each other. c) Experimental spectra of polycrystalline RuO_2 normalized to the edge jump at 551.15 eV and plotted with a y-offset. The green dotted line shows the shift of the peak towards lower excitation energy with the increase in applied potential.*

S7 Reversibility of deprotonation reaction (from O K-edge XAS)

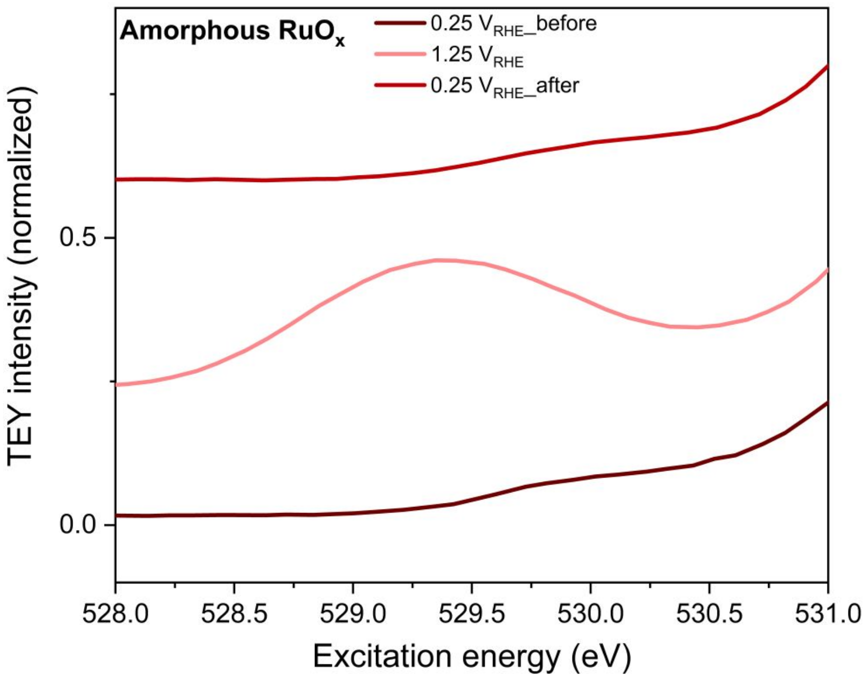

Figure S7: Reversibility of the deprotonation events observed in O K-edge XAS.

S8 Ex-situ Ru M_3-edge XAS

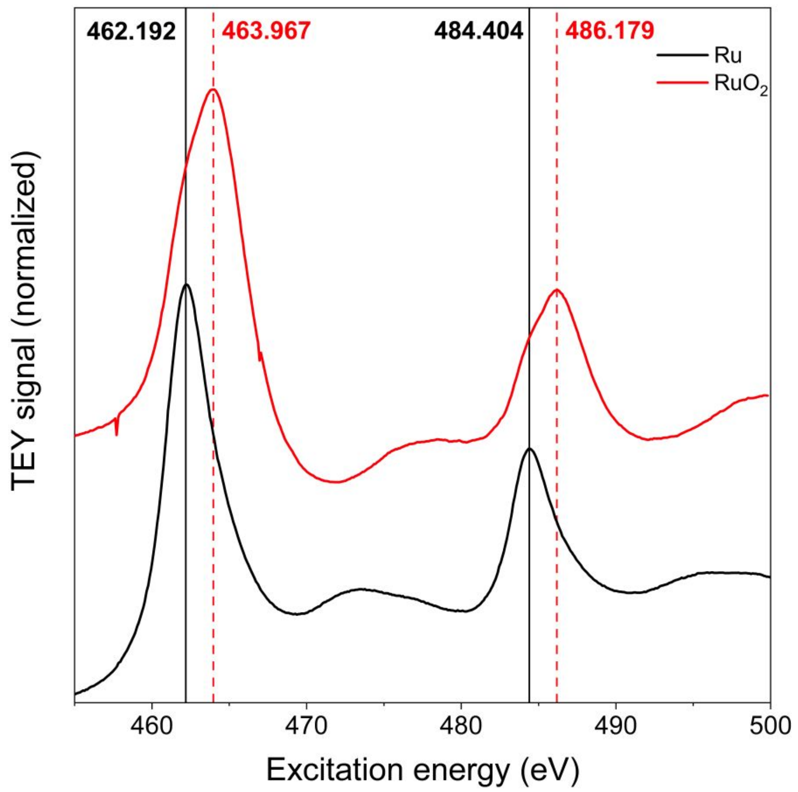

Figure S8: Ex situ Ru M_3-edge XAS of Ru and RuO_2.

S9 Ruthenium $L_{3,2}$-edge XAS

Ru $L_{3,2}$-edge spectroscopy is a powerful tool to study Ru oxidation states. Since the spectra could only be recorded in fluorescence yield mode XAS, thicker catalyst layers were required to obtain sufficient signal. Therefore, we used a hydrous RuO_2.xH2O powder as our amorphous ruthenium oxide sample for Ru $L_{3,2}$-edge spectroscopy instead of the very thin electrochemically oxidized RuO_x layer using in the IEM cell. The powder was investigated using tender X-rays at the Ru $L_{3,2}$-edge at while the electrode was held at several potentials. The ex-situ Ru $L_{3,2}$-edge spectrum of the hydrous powder is shown in figure S9 a. The Ru L_3 white line occurs at approximately 2841.41 eV followed by L_2 white line at approximately 2970 eV, which could be attributed to electronic transitions corresponding to $2p_{3/2}\rightarrow 4d$ and $2p_{1/2}\rightarrow 4d$ orbitals respectively. Each white line is followed by a step edge associated to 2p→ continuum electronic excitations, with some weak features due to multiple backscattering of photoelectrons from neighboring atoms.

The raw data has been "unit-edge step normalized" at the L_3 edge which results in L_2 absorption edge normalized to 0.5 according to the procedure described elsewhere[8]. Two arctangent functions each having heights of 1 and 0.5 and centered at the inflection point has been used to model the continuum edge step at the L_3 and L_2 absorption edges respectively as represented by the red curve in figure S9 a. In $RuO_2.xH_2O$, ruthenium ions exist in a tetragonally distorted RuO_6 octahedral coordination where Ru 4d orbitals are split into partially filled t_{2g} bands with 4 electrons and empty e_g bands[6]. The crystal field splitting (CFT) of the 4d orbital into t_{2g} and e_g orbitals is indeed visible on a close inspection of the $L_{3,2}$ white line (figure S9 b-c) which shows that both L_3 and L_2 white lines contain a doublet that could not be resolved in the $M_{3,2}$ edge. The holes at the 2p level are deeper and have higher binding energy as compared to 3p holes which results in a longer lifetime for 2p holes thereby giving rise to a well resolved doublet structure in the L-edge as compared to M-edge spectra[9].

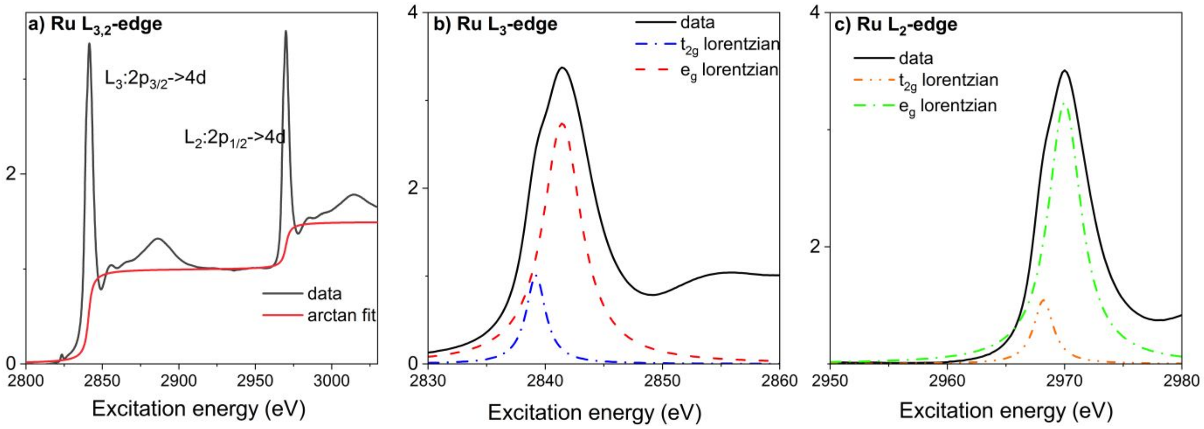

Figure S9: Ex-situ Ru L-edge XAS. *a) Ru L_3 and L_2 edge of commercially bought $RuO_2.xH_2O$ powder. The red line represents the fitted arc tan function to capture the edge jump, b-c) Two lorentzian peaks fitted into Ru L_3 and L_2 edge to denote the t_{2g} and e_g contribution arising due to crystal field splitting.*

The as-purchased $RuO_2.xH_2O$ powder is expected to have a Ru^{4+} oxidation state with most of the bridging oxygen sites deprotonated during the ex-situ vacuum measurement. When exposed to aqueous electrolyte, the undercoordinated surface oxygen atoms should undergo protonation leading to heavily hydrated RuO_6 octahedral units. In this state, majority of the ruthenium centers are expected to exist in the Ru^{3+} oxidation state. This is indeed evident from the Ru L edge spectra at 0.1 V_{RHE} with the L_3 and L_2 white line peak position at approximately 2841.19 eV and 2969.9 eV, respectively. A very weak shoulder is observed in the L_3 edge whereas no doublet is observed at the L_2 edge. This is in line with the report by Sham et al.[10] who measured the $L_{3,2}$ edge spectra of Ru^{3+} in $Ru(NH_3)_6Cl_6$ and observed a doublet at the L_3 edge and a single peak at L_2 edge. The presence of the doublet at L_3 edge and a single peak at L_2 edge for Ru^{3+} compounds is attributed to the J selection rules which forbids the $2p_{1/2} \rightarrow 4d_{5/2}$ transition at the L_2 edge. This phenomenon has been explained quantitatively through crystal field multiplet calculations by Groot et al.[11] and further confirmed for $RuCl_3$ by Plumb et al.[12]

The operando measurements (figure S10 a) show that the Ru L_3 white line resonance intensity increases and the peak position shifts towards higher energies with the increase in applied

potential. This indicates an increase in the number of 4d holes and hence oxidation of the Ru site. The L_3 white line peak position in ruthenium is determined by the electrostatic attraction between the nucleus and the 2p electrons. This coulombic interaction is shielded by the rest of the electrons. At a higher oxidation state, the effective charge of the nucleus increases due to decrease in shielding which results in the requirement of more energetic X-rays to excite the 2p electron to the unoccupied 4d levels. The L_3 white line peak position at 1.2 V_{RHE} is at 2842.4 eV which indicates that the majority of the ruthenium centers exist in Ru^{4+} oxidation state just before the onset of OER. This is evident from the measurements by Kim et al.[13] who found the white line peak position for Ru^{4+} and Ru^{5+} perovskite compounds to be approximately 2843 eV and 2844.5 eV respectively.

The doublet nature of the L_3 white line can be visualized through a second derivative analysis of the peaks (figure S10 b) which illustrates a difference in the CFT. At the L_3 edge, the t_{2g} peak position shifts from 2839.4 eV to 2840 eV and the e_g peak shifts from 2841 eV to 2842.4 eV. This causes the energy difference between the t_{2g} and e_g transitions, ($\Delta E_{(eg\text{-}t2g)}$), to increase from 1.6 to 2.4 when the applied potential is increased from 0.1 V_{RHE} to 1.2 V_{RHE}. A similar behavior of reduced crystal field splitting or diminishing t_{2g} shoulder in the rising edge of L_3 white line with the increase in water content of the sample has been reported by Dmowski et al.[14] The value of ($\Delta E_{(eg\text{-}t2g)}$) ~2.4 has been reported for Ru^{4+} compounds by Wu et al.[15] and rutile RuO_2 microcrystals by Zhou et al.[16] ($\Delta E_{(eg\text{-}t2g)}$) values of greater than 3.2 are expected for Ru^{5+} compounds as reported by Kim et al.[13] Hence, the CFT analysis confirms the analysis from the peak positions of both the Ru L-edge and Ru M-edge data, indicating a $Ru^{3+} \rightarrow Ru^{4+}$ transition as the sample is oxidized.

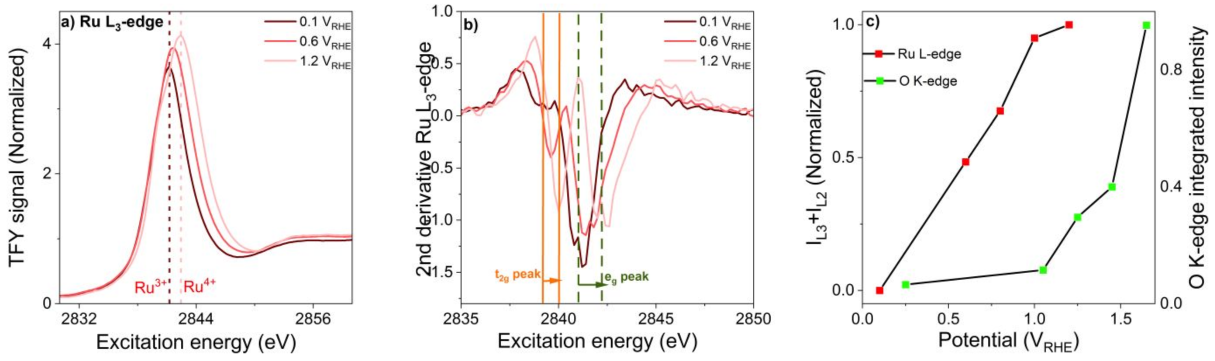

Figure S10: Operando Ru L-edge XAS. *a) Ru L_3-edge shifts to higher excitation energy with increase in applied potential, b) 2nd derivative of Ru L_3-edge showing the t_{2g} peak and e_g peak, c) Comparison of potential dependence of total integrated intensity ($I_{L3}+I_{L2}$) from Ru L-edge XAS with potential dependence of undercoordinated oxygen species (μ_1-O and μ_2-O) of amorphous ruthenium oxide from O K-edge XAS.*

In 4d transition elements, the sum of the integrated intensity of the L_3 and L_2 peaks ($I_{L3}+I_{L2}$) is directly proportional to the local density of unoccupied 4d states or the number of 4d holes in the system in ground state[17]. Thus, ($I_{L3}+I_{L2}$) data is also an oxidation state indicator. It can be seen in the red plot figure S10 c that the ($I_{L3}+I_{L2}$) increases almost linearly with increase in applied potential. This verifies the fact that the ruthenium oxidation state increases with applied potential, in agreement with the Ru M-edge data. However, beyond 1.0 V_{RHE} the slope of ($I_{L3}+I_{L2}$) appears to be decreasing which hints towards saturation of Ru oxidation. The saturation of Ru oxidation states at high overpotentials indicates that the ruthenium sites do not undergo drastic oxidation beyond Ru^{4+} just before the onset of OER, in agreement with the Ru M_3-edge data.

References:

(1) Nilsson, A.; Nordlund, D.; Waluyo, I.; Huang, N.; Ogasawara, H.; Kaya, S.; Bergmann, U.; Näslund, L. Å.; Öström, H.; Wernet, P.; Andersson, K. J.; Schiros, T.; Pettersson, L. G. M. X-Ray Absorption Spectroscopy and X-Ray Raman Scattering of Water and Ice; an Experimental View. *J. Electron Spectros. Relat. Phenomena* **2010**, *177* (2–3), 99–129. https://doi.org/10.1016/j.elspec.2010.02.005.

(2) Velasco-Velez, J. J.; Wu, C. H.; Pascal, T. A.; Wan, L. F.; Guo, J.; Prendergast, D.; Salmeron, M. The Structure of Interfacial Water on Gold Electrodes Studied by X-Ray Absorption Spectroscopy. *Science (80-.).* **2014**, *346* (6211), 831–834. https://doi.org/10.1126/science.1259437.

(3) Frati, F.; Hunault, M. O. J. Y.; De Groot, F. M. F. Oxygen K-Edge X-Ray Absorption Spectra. *Chemical Reviews*. 2020, pp 4056–4110. https://doi.org/10.1021/acs.chemrev.9b00439.

(4) Fransson, T.; Harada, Y.; Kosugi, N.; Besley, N. A.; Winter, B.; Rehr, J. J.; Pettersson, L. G. M.; Nilsson, A. X-Ray and Electron Spectroscopy of Water. *Chemical Reviews*. 2016, pp 7551–7569. https://doi.org/10.1021/acs.chemrev.5b00672.

(5) Aarva, A.; Deringer, V. L.; Sainio, S.; Laurila, T.; Caro, M. A. Understanding X-Ray Spectroscopy of Carbonaceous Materials by Combining Experiments, Density Functional Theory, and Machine Learning. Part II: Quantitative Fitting of Spectra. *Chem. Mater.* **2019**, *31* (22), 9256–9267. https://doi.org/10.1021/acs.chemmater.9b02050.

(6) Over, H. Surface Chemistry of Ruthenium Dioxide in Heterogeneous Catalysis and Electrocatalysis: From Fundamental to Applied Research. *Chemical Reviews*. 2012, pp

3356–3426. https://doi.org/10.1021/cr200247n.

(7) Mom, R. V.; Falling, L. J.; Kasian, O.; Algara-Siller, G.; Teschner, D.; Crabtree, R. H.; Knop-Gericke, A.; Mayrhofer, K. J. J.; Velasco-Vélez, J. J.; Jones, T. E. Operando Structure-Activity-Stability Relationship of Iridium Oxides during the Oxygen Evolution Reaction. *ACS Catal.* **2022**, *12* (9), 5174–5184. https://doi.org/10.1021/acscatal.1c05951.

(8) Clancy, J. P.; Chen, N.; Kim, C. Y.; Chen, W. F.; Plumb, K. W.; Jeon, B. C.; Noh, T. W.; Kim, Y. J. Spin-Orbit Coupling in Iridium-Based 5d Compounds Probed by x-Ray Absorption Spectroscopy. *Phys. Rev. B - Condens. Matter Mater. Phys.* **2012**, *86* (19), 195131. https://doi.org/10.1103/PhysRevB.86.195131.

(9) Lebert, B. W.; Kim, S.; Bisogni, V.; Jarrige, I.; Barbour, A. M.; Kim, Y. J. Resonant Inelastic X-Ray Scattering Study of α-$RuCl_3$: A Progress Report. *J. Phys. Condens. Matter* **2020**, *32* (14), 144001. https://doi.org/10.1088/1361-648X/ab5595.

(10) Sham, T. K. X-Ray Absorption Spectra of Ruthenium L Edges in $Ru(NH_3)_6Cl_3$. *J. Am. Chem. Soc.* **1983**, *105* (8), 2269–2273. https://doi.org/10.1021/ja00346a028.

(11) De Groot, F. M. F.; Hu, Z. W.; Lopez, M. F.; Kaindl, G.; Guillot, F.; Tronc, M. Differences between L_3 and L_2 X-Ray Absorption Spectra of Transition Metal Compounds. *J. Chem. Phys.* **1994**, *101* (8), 6570. https://doi.org/10.1063/1.468351.

(12) Plumb, K. W.; Clancy, J. P.; Sandilands, L. J.; Shankar, V. V.; Hu, Y. F.; Burch, K. S.; Kee, H. Y.; Kim, Y. J. α- $RuCl_3$: A Spin-Orbit Assisted Mott Insulator on a Honeycomb Lattice. *Phys. Rev. B - Condens. Matter Mater. Phys.* **2014**, *90* (4), 041112. https://doi.org/10.1103/PhysRevB.90.041112.

(13) Kim, J. Y.; Hwang, S. H.; Kim, S. J.; Demazeau, G.; Choy, J. H.; Shimada, H. 4d

Electronic Structure Analysis of Ruthenium in the Perovskite Oxides by Ru K- and L-Edge XAS. *J. Synchrotron Radiat.* **2001**, *8* (2), 722–724. https://doi.org/10.1107/S0909049500017787.

(14) Dmowski, W.; Egami, T.; Swider-Lyons, K. E.; Yan, W. F.; Dai, S.; Overbury, S. H. Local Atomic Structure in Disordered and Nanocrystalline Catalytic Materials. *Zeitschrift fur Krist.* **2007**, *222* (11), 617–624. https://doi.org/10.1524/zkri.2007.222.11.617.

(15) Wu, H. H.; Chen, S. W.; Lin, B. N.; Hsu, Y. Y.; Lee, J. F.; Jang, L. Y.; Ku, H. C. Ru L_3-Edge XANES Studies of A_2RuO_4 and $ARuO_3$ (A = Ca, Sr, Ba) Ruthenates. *J. Low Temp. Phys.* **2003**, *131* (5–6), 1193–1197. https://doi.org/10.1023/A:1023434017456.

(16) Zhou, J. G.; Fang, H. T.; Hu, Y. F.; Sham, T. K.; Wu, C. X.; Liu, M.; Li, F. Immobilization of RuO_2 on Carbon Nanotube: An X-Ray Absorption near-Edge Structure Study. *J. Phys. Chem. C* **2009**, *113* (24), 10747–10750. https://doi.org/10.1021/jp902871b.

(17) Wang, H.; Ge, P.; Riordan, C. G.; Brooker, S.; Woomer, C. G.; Collins, T.; Melendres, C. A.; Graudejus, O.; Bartlett, N.; Cramer, S. P. Integrated X-Ray L Absorption Spectra. Counting Holes in Ni Complexes. *J. Phys. Chem. B* **1998**, *102* (42), 8343–8346. https://doi.org/10.1021/jp9821026.
